# Association between life events, anxiety, depression and non-suicidal self-injury behavior in Chinese psychiatric adolescent inpatients: a cross-sectional study

**DOI:** 10.3389/fpsyt.2023.1140597

**Published:** 2023-07-03

**Authors:** Qingqing Xiao, Xiaozhen Song, Lijuan Huang, Dandan Hou, Xuehua Huang

**Affiliations:** ^1^Mental Health Center, West China Hospital, Sichuan University, Chengdu, China; ^2^West China School of Nursing, Sichuan University, Chengdu, China

**Keywords:** non-suicidal self-injury, life events, anxiety, depression, psychiatric adolescent inpatients

## Abstract

**Background:**

Non-suicidal self-injury (NSSI) is a major public health concern among adolescents. Further research is needed into contributors to this behavior, in particular among adolescents with psychiatric disorders. The aim of the present study was to explore the impact of life events and emotional stress on NSSI among hospitalized psychiatric adolescents.

**Methods:**

In this cross-sectional study, 505 Chinese psychiatric adolescent inpatients 10–19  years old completed questionnaires about sociodemographic characteristics and NSSI as well as the Adolescent Self-Rating Life Events Checklist (ASLEC), the State–Trait Anxiety Inventory Form Y, and the Center for Epidemiological Studies Depression Scale. Chi-square test was used to compare the incidence of NSSI in psychiatric adolescent patients with different sociodemographic. *T*-test was used to compare the total scores and dimension scores of the ASLEC, STAI-Y, and CES-D between the NSSI group and the non-NSSI group. A binary logistic regression model was built to explore the relationships among sociodemographic characteristics, questionnaire scores and NSSI.

**Results:**

Most psychiatric adolescent inpatients (393, 77.8%) reported NSSI behavior. The higher risk for NSSI was observed among female (odds ratio [OR] 2.665, 95% confidence interval [CI] 1.575–4.510), younger adolescents (10–14 years; OR 2.021, 95% CI 1.258–3.245), with a suicide history (OR 2.479, 95% CI 1.549–3.967), or with depression symptom (OR 3.217, 95% CI 1.572–6.582) and those with higher scores of ASLEC (OR 1.019, 95% CI 1.010–1.029).

**Conclusion:**

Our study in China is one of the first to apply to adolescent inpatients the diagnostic criteria of NSSI in the latest edition of the *Diagnostic and Statistical Manual of Mental Disorders*. Our analysis suggests that NSSI prevalence is disturbingly high among adolescents with mental illness in China. A better understanding of contributing factors, especially negative life events and negative emotions, may guide interventions that can reduce its prevalence.

## Introduction

Non-suicidal self-injury (NSSI) is defined as the direct and deliberate destruction of one’s own body tissue for socially unacceptable purposes but without suicidal intent (1). The most common forms of NSSI are cutting, scratching, hitting, carving, and scraping oneself (2). NSSI serves various functions that are not mutually exclusive, including affect regulation, reduction of mental pain, transfer of mental pain onto the body, self-punishment, influence over other people, anti-dissociation, anti-suicide, and thrill-seeking (3).

Adolescence is a special stage of development, in this period, the individual’s physical development is rapid, but the speed of psychological development is relatively lagging behind, often appear a series of emotional and behavioral problems, the growth and development of adolescents have a negative impact (4). Some studies have shown that NSSI often first appears in adolescence, and at the same time appears with emotional problems, which may be a prelude to suicide (5, 6). Among adolescents, NSSI is strongly associated with mental health problems (7, 8). In fact, risk of suicidal ideation among adolescents increases with the number of forms of NSSI that they practice (9). NSSI is particularly prevalent among adolescents 15–16 years old, making it a major concern for child and adolescent psychiatrists and psychotherapists (10).

Studies in several countries have reported that 9–44% of adolescents engaged in at least one form of NSSI within the preceding 12 months (11–14). A recent meta-analysis involving 264,638 adolescents in nonclinical populations reported a lifetime NSSI prevalence of 22% and 12-month prevalence of 23% (15). NSSI is even more prevalent among psychiatric inpatients (16, 17). A German survey showed that 40–60% psychiatric inpatients report at least one NSSI episode during their lives, and 50% report recurring NSSI (16). A survey in China showed that 62% of psychiatric inpatients with depression or bipolar disorder at two psychiatric hospitals in China reported having engaged in NSSI during the previous year (17).

Despite its prevalence, NSSI has traditionally been studied and treated only as a symptom of borderline personality disorder. This has begun to change with the release of the 5th edition of the *Diagnostic and Statistical Manual of Mental Disorders* (DSM-5) (18). In the general population, according to DSM-5 criteria, NSSI prevalence ranges from around 1.5 to 6.7% of children and adolescents (2). But among inpatients, only a few studies have researched the prevalence and risk factors of NSSI behavior based on the diagnostic criteria recommended by DSM-5 (6, 19, 20). In China, such studies are rare in psychiatric wards. Little studies have applied these new diagnostic criteria for NSSI to adolescents with psychiatric disorders (21). To underpin the clinical relevance and utility more data from the clinical field is warranted (22).

Given the paucity of evidence-based treatments for NSSI in adolescents with mental disorders, we need further research into the factors that contribute to it (23), particularly within the framework of the new DSM-5 criteria. The related factors of adolescents NSSI are complex, many risk factors for NSSI have been proposed (17, 18, 24), especially negative life events, depression, anxiety, and other emotional symptoms (25–27). Symptoms of depression and anxiety serve as negative indicators of mental health, which is an important problem especially during adolescence where incidence rates are high (28), which has become an important influence factor of NSSI (29, 30). Negative life events refer to events that an individual feels unpleasant. These events mostly come from changes and stimuli in the individuals’ family, learning environment, which may cause negative psychological and physiological outcomes, and cause great damage to physical and mental health (31). Stressful life events (such as family conflicts, conflict with teachers, classmate disputes, economic distress, death of relatives, and failure in examination) increase the risk of self-harm, particularly in the form of suicidal ideation and behavior (23, 26). Nock’s integrated theoretical model of NSSI pointed that NSSI is an effective means of regulating social situations, which has a function of interpersonal negative reinforcement (32). And stressful life events was linked to NSSI because of deviant peer affiliation (33). Even though some researchers point out that negative life events are an important factor in the survey of NSSI, but few have clarified the relationship between NSSI in adolescent psychiatric inpatients with negative life events. At the same time, in China, not only are there few studies on negative life events and NSSI in adolescents, but small-scale samples (27). Therefore, this study plan to apply the DSM-5 criteria to assess the influence of life events, anxiety, and depression on NSSI among Chinese adolescent psychiatric inpatients. We assume that in this sample, compared with the adolescent psychiatric inpatients in the non-NSSI group, the NSSI group experienced more negative life events and had more obvious anxiety and depression symptoms.

## Methods

### Participants

In this study, 520 adolescents with psychiatric mental illness were recruited by convenience sampling from 21 September 2020 to 4 November 2021 in the Mental Health Center of the Psychiatric Department at West China Hospital of Sichuan University (Chengdu, Sichuan, China). Participants met the following criteria: (a) psychiatric inpatients with any type of psychiatric disorder; (b) 10–19 years old; (c) relatively stable emotional state and normal abilities in reading, comprehension and expression, as well as willingness to complete the paper-and-pencil tests; and (d) willingness to participate in the study. The exclusion criteria were (a) multiple registration and foreign nationality data; or (b) serious physical disease and/or nervous system disease. This study was approved by the Biomedical Research Ethics Committee, West China Hospital of Sichuan University (approval 20201049). All adolescents and their legal guardians provided written informed consent.

### Questionnaires

#### Sociodemographic and clinical characteristics

A self-administered questionnaire was established to collect sociodemographic data on gender, age, school grade, registered residence, only-child status (yes/no), family structure, educational level of parents, family economic status, parental expectations, frequency of scolding by parents, frequency of corporal punishment by parents, family history of mental illness, in charge of the class (yes/no), academic record, learning burden, first hospitalization or not, current main diagnosis, and history of suicide attempts (yes/no).

#### Non-suicidal self-injury questionnaire

The use of single-item measure is common in the NSSI literature indicated that the prevalence of NSSI does not significantly differ across the different methods used (i.e., single-item or checklist method) (34). Referring to the relevant research (12, 35), this study adopted the single-item method to evaluate NSSI. To determine the prevalence of NSSI, each participant was asked the following question: “Adolescents have to deal with a lot of stress. During the past year, when you had problems to deal with, did you ever physically hurt yourself on purpose?.” Follow-up questions asked specifically whether the self-injury took any of the following forms: beating, hair-pulling, hitting the head or fist against other objects, pinching or scratching, biting, cutting, stabbing, drug overdose, drinking or smoking, or deliberately swallowing foreign objects. The number of occurrences of each type of behavior during the previous year was recorded. Based on the DSM-5, we defined NSSI disorder as having engaged in more than five NSSI behaviors during the past year (36).

#### Adolescent self-rating life events checklist

The adolescent self-rating life events checklist (ASLEC) (31) was used to assess the frequency and intensity of negative life events during the past 12 months that may have affected the respondent psychologically. The survey consists of 27 items across six subscales, including interpersonal relationships, study pressure, punishment, sense of loss, healthy adaptation, and other factors. Participants responded to each item on a six-point Likert scale from 0 (did not occur) to 5 (extremely severe), with higher score indicating greater stress. In this study, the Cronbach value of the ASLEC scale was 0.8492.

#### State–trait anxiety inventory form Y

The State–Trait Anxiety Inventory Form Y (STAI-Y) is a self-report instrument testing state anxiety and trait anxiety. State anxiety is a temporary emotional state defined mainly by present feelings. Trait anxiety, in contrast, is a relatively stable response to stressful situations that involves persistent, general feelings of anxiety. The Chinese version of the STAI-Y contains 40 items, half of them assessing state anxiety and the other half measuring trait anxiety (37). Participants responded to all items on a scale from 1 (not at all) to 4 (very much so). The total score of the STAI-Y was the sum of all items, with higher score indicating higher anxiety level. Cronbach’s alpha has been reported as 0.84 for state anxiety and 0.87 for trait anxiety (38).

#### Center for epidemiological studies depression scale

The center for epidemiological studies depression scale (CES-D) is a 20-item, self-report scale that measures depressive symptoms during the previous week (39). We used the originally recommended cutoff score of ≥ 16 on the CES-D to identify those with depression (39). In a recent meta-analysis, this cutoff showed sensitivity of 0.87 and specificity of 0.70 for detecting depression in the general population (40). The CES-D has four subscales: depressed affect, somatic symptoms, interpersonal problems, and positive affect.

### Data collection

After explaining the purpose and significance of the study, we administered the questionnaires in a relatively quiet place. We read the instructions to the participants, then invited them to complete the forms themselves. A total of 520 questionnaires were distributed, of which 505 were retained after eliminating damaged or unclear questionnaires.

### Data analysis

Sociodemographic data were expressed as frequencies and percentages (*n*, %), while scores on the ASLEC, STAI-Y, and CES-D were reported as mean ± standard deviation (SD). Chi-square test was used to compare the incidence of NSSI in psychiatric adolescent patients with different sociodemographic. *T*-test was used to compare the total scores and dimension scores of the ASLEC, STAI-Y, and CES-D between the NSSI group and the non-NSSI group.

Risk factors for NSSI in our sample were identified using binary logistic regression, in which the dependent variable was occurrence of NSSI and the independent variables were factors that were statistically significant in univariate analysis. For the ordered variables, when the direct substitution into the regression equation was meaningless or the doubt effect did not show an equidistant and proportional relationship among the grades, the dummy variables were processed, and then the regression analysis was carried out. All the variables were entered in the model using the forward Wald method. All statistical analyses were performed in SPSS 21 (IBM Corp., Armonk, NY, United States), and results associated with *p* < 0.05 were considered statistically significant.

## Results

### Clinicodemographic characteristics of the study population

Our final analysis included 505 adolescents (404 females), who were an average of 14.54 ± 1.75 years old (range, 10–19 years; Table 1). A total of 77.82% (*n* = 393) of the participants had NSSI behavior. A total of 325 (64.4%) patients came from urban areas, 243 (48.1%) patients came from nuclear families, and 222 (44.0%) were the only child in the family. A few patients (95, 18.8%) believed that the family economic status was poor. Many patients 208 (41.2%) believed that parental expectation was high, even 97 (19.2%) patients thought that parents’ expectation was extremely high. More than half (310, 61.4%) thought their learning burden was heavy. Only 47 (9.3%) adolescents had a family history of suicide, but most (305, 60.4%) reported having attempted it themselves. Most adolescents (333, 65.9%) were being admitted to the psychiatric ward for the first time, and 309 (61.2%) adolescents were diagnosed with anxiety and depression, 22 (4.4%) with schizophrenia, 55 (10.9%) with childhood emotional disorder, 66 (13.1%) with bipolar emotional disorder, and 76 (15.0%) with other mental disorders. Gender, age, school grade, parental scolding, learning burden, main psychiatric diagnosis, suicide history had significant differences in the influence of NSSI behavior among psychiatric hospitalized adolescents (*p* < 0.05).

**Table 1:** Comparison of demographic and clinical characteristics of Chinese adolescents hospitalized in a psychiatric department with or without non-suicidal self-injury (NSSI) behavior (*n* = 505).

Variable	Total Sample *n* (%)	NSSI group *n* (%)	Non-NSSI group *n* (%)	***χ*** ^**2**^	***p***
Gender					
Male	101 (20.0)	61 (60.4)	40 (39.6)	22.212	**<0.001**
Female	404 (80.0)	332 (82.2)	72 (17.8)		
Age, years					
10–14	268 (53.1)	223 (83.2)	45 (16.8)	9.602	**0.002**
15–19	237 (46.9)	170 (71.7)	67 (28.3)		
School grade					
Junior high school and below	294 (58.2)	239 (81.3)	55 (18.7)	4.911	**0.027**
High school and above	211 (41.8)	154 (73.0)	57 (27.0)		
Only child					
Yes	222 (44.0)	176 (79.3)	46 (20.7)	0.488	0.485
No	283 (56.0)	217 (76.7)	66 (23.3)		
Registered residence					
Urban	325 (64.4)	256 (78.8)	69 (21.2)	0.474	0.491
Rural	180 (35.6)	137 (76.1)	43 (23.9)		
Family structure					
Nuclear family	243 (48.1)	186 (76.5)	57 (23.5)	0.209	0.648
Nonnuclear family	262 (51.9)	205 (78.2)	57 (21.8)		
Father’s education level					
Junior high school and below	201 (39.8)	153 (76.1)	48 (23.9)	2.027	0.363
High school or technical secondary school	125 (24.8)	103 (82.4)	22 (17.6)		
College degree or above	179 (35.4)	137 (76.5)	42 (23.5)		
Mother’s education level					
Junior high school and below	239 (47.3)	186 (77.8)	53 (22.2)	1.446	0.485
High school or technical secondary school	122 (24.2)	99 (81.1)	23 (18.9)		
College degree or above	144 (28.5)	108 (75.0)	36 (25.0)		
Family economic status					
Poor	95 (18.8)	77 (81.1)	18 (18.9)	2.059	0.357
Medium	294 (58.2)	231 (78.6)	63 (21.4)		
Good	116 (23.0)	85 (73.3)	31 (26.7)		
Parental expectations					
Extremely low	18 (3.6)	16 (88.9)	2 (11.1)	8.233	0.083
Low	26 (5.1)	22 (84.6)	4 (15.4)		
Medium	156 (30.9)	110 (70.5)	46 (29.5)		
High	208 (41.2)	169 (81.3)	39 (18.8)		
Extremely high	97 (19.2)	76 (78.4)	21 (21.6)		
Scolded by parents					
Never	18 (3.56)	12 (66.7)	6 (33.3)	7.995	**0.018**
Occasionally or Sometimes	277 (54.8)	205 (74.0)	72 (26.0)		
Often or always	210 (41.6)	176 (83.8)	34 (16.2)		
Corporal punishment by parents					
Never	185 (36.6)	134 (72.4)	51 (27.6)	4.984	0.083
Occasionally or sometimes	269 (53.3)	217 (80.7)	52 (19.3)		
Often or always	51 (10.1)	42 (82.4)	9 (17.6)		
Family history of suicide					
Yes	47 (9.3)	37 (78.7)	10 (21.3)	0.024	0.876
No	458 (90.7)	356 (77.7)	102 (22.3)		
Family history of mental illness					
Yes	83 (16.4)	61 (73.5)	22 (26.5)	1.078	0.299
No	422 (83.6)	332 (78.7)	90 (21.3)		
In charge of the class					
Yes	267 (52.9)	206 (77.2)	61 (22.8)	0.147	0.702
No	238 (47.1)	187 (78.6)	51 (21.4)		
Academic records					
Poor	188 (18.02)	151 (80.3)	37 (19.7)	2.393	0.664
Medium	167 (33.07)	128 (76.6)	39 (23.4)		
Good	150 (24.16)	114 (76.0)	36 (24.0)		
Learning burden					
Light	69 (13.7)	42 (60.9)	27 (39.1)	14.070	**0.001**
Medium	126 (24.9)	98 (77.8)	28 (22.2)		
Heavy	310 (61.4)	253 (81.6)	57 (18.4)		
First hospitalization					
Yes	333 (65.9)	263 (79.0)	70 (21.0)	0.759	0.384
No	172 (34.1)	130 (75.6)	42 (24.4)		
Current main diagnosis					
Anxiety and depression	309 (61.2)	253 (81.9)	56 (18.1)	15.088	**0.005**
Schizophrenia	22 (4.4)	16 (72.7)	6 (27.3)		
Childhood emotional disorder	55 (10.9)	36 (65.5)	19 (34.5)		
Bipolar disorder	66 (13.1)	58 (87.9)	8 (12.1)		
Other	76 (15.0)	53 (69.7)	23 (30.3)		
Ever tempted suicide					
Yes	305 (60.4)	263 (86.2)	42 (13.8)	31.542	**<0.001**
No	200 (39.6)	130 (65.0)	70 (35.0)		

Differences assessed by Chi-square test.

### Psychological assessment and NSSI

Table 2 showed that compared to the non-NSSI group, the NSSI group showed significantly higher total ASLEC score, and the score of interpersonal relationships, study pressure, punishment, healthy adaptation, and other factors showed statistically significant positively correlated with NSSI prevalence (all *p* < 0.01), but there was no significant difference in the score of sense of loss between the two groups (*p* > 0.05). The STAI-Y scale showed that the state anxiety, trait anxiety scores of adolescent psychiatric patients with NSSI was significantly higher than that of patients without NSSI (all *p* < 0.001). We also found that there were higher score for CES-D in the NSSI group than the non-NSSI group. Table 3 displayed that the NSSI group also showed significantly higher rates of anxiety and depression (all *p* < 0.001).

**Table 2:** Comparison of total and dimension scores on questionnaires between the non-suicidal self-injury (NSSI) group and non-NSSI group (*n* = 505) by Student’s *t*-test.

Scale/dimension	NSSI group (mean ± SD)	Non-NSSI group (mean ± SD)	*t*	95% CI	*p*
ASLEC					
Interpersonal relationship	16.89 ± 6.268	12.46 ± 6.613	6.512	(3.091, 5.762)	**<0.001**
Study pressure	14.5 3 ± 5.842	11.11 ± 5.861	5.469	(2.194, 4.655)	**<0.001**
Punishment	15.60 ± 8.158	11.76 ± 8.095	4.404	(2.128, 5.556)	**<0.001**
Sense of loss	4.87 ± 3.912	4.15 ± 3.783	1.721	(−0.101, 1.533)	0.086
Healthy adaptation	9.71 ± 4.422	7.41 ± 4.513	4.837	(1.367, 3.237)	**<0.001**
Other factors	10.19 ± 4.399	7.19 ± 4.749	6.261	(2.061, 3.946)	**<0.001**
Total score	71.79 ± 27.308	54.08 ± 27.798	6.032	(11.944, 23.483)	**<0.001**
STAI					
State anxiety	61.15 ± 11.675	52.09 ± 13.588	6.416	(6.274, 11.853)	**<0.001**
Trait anxiety	59.55 ± 9.677	51.43 ± 11.601	6.770	(5.753, 10.494)	**<0.001**
*CES-D*	41.06 ± 12.291	28.59 ± 14.319	8.378	(9.530, 15.408)	**<0.001**

ASLEC, adolescent self-rating life events checklist; CES-D, center for epidemiological studies depression scale; CI, confidence interval; OR, odds ratio.CI, confidence interval; SD, standard deviation.

**Table 3:** Comparison of the rates of anxiety and depression between the non-suicidal self-injury (NSSI) group and non-NSSI group (*n* = 505).

Variable	Total Sample *n* (%)	NSSI group *n* (%)	Non-NSSI group *n* (%)	***χ*** ^**2**^	***p***
State anxiety					
Yes	463 (91.7)	373 (80.6)	90 (19.4)	24.212	**<0.001**
No	42 (8.3)	20 (47.6)	22 (52.4)		
Trait anxiety					
Yes	473 (93.7)	379 (80.1)	94 (19.9)	22.980	**<0.001**
No	32 (6.3)	14 (43.8)	18 (56.3)		
Depression					
Yes	459 (90.9)	373 (81.3)	86 (18.7)	34.586	**<0.001**
No	46 (9.1)	20 (43.5)	26 (56.5)		

Differences assessed by Chi-square test.

### Factors influencing NSSI behavior

Binary logistic regression identified the following factors as differing significantly between respondents with or without NSSI: age, gender, school grade, parental scolding, learning burden, main psychiatric diagnosis, suicide history, state anxiety, trait anxiety, depression, and life events.

These variables were integrated into multivariate regression as independent variables, while the ordinal variables of parental scolding, learning burden and main diagnosis were represented by dummy variables. The assignment method of the argument was as follows: gender: male = 0, female = 1; age: 15–19 years = 0, 10–14 years = 1; grade: Junior high school and below = 0, High school and above = 1; suicide history: no = 0, yes = 1; state anxiety: no = 0, yes = 1; trait anxiety: no = 0, yes = 1; CES-D: no = 0, yes = 1. Dummy variables were assigned as follows: scolded by parents: never = 0, occasionally or sometimes/often or always = 1; learning burden: light = 0, medium/heavy = 1, main psychiatric diagnosis: anxiety/depression = 0, schizophrenia/childhood emotional disorder/bipolar disorder/others = 1.

The dependent variable was whether the adolescents was diagnosed with NSSI according to the DSM-5 criteria. Age, gender, suicide history, depression, and ASLEC score remained associated with NSSI in the multivariate analysis. The higher risk for NSSI was observed among female (odds ratio [OR] 2.665, 95% confidence interval [CI] 1.575–4.510), younger adolescents (10–14 years; OR 2.021, 95% CI 1.258–3.245), with a suicide history (OR 2.479, 95% CI 1.549–3.967), or with depression symptom (OR 3.217, 95% CI 1.572–6.582) and those with higher scores of ASLEC (OR 1.019, 95% CI 1.010–1.029; Table 4).

**Table 4:** Logistic regression to identify factors influencing non-suicidal self-injury (NSSI) behavior in psychiatric adolescent inpatients (*n* = 505).

Independent variable	*β* value	Standard error	Wald	*OR*	95% CI	*p*
Gender	0.980	0.268	13.341	2.665	(1.575, 4.510)	**<0.001**
Age	0.703	0.242	8.466	2.021	(1.258, 3.245)	**0.004**
CES-D	1.168	0.365	10.230	3.217	(1.572, 6.582)	**0.001**
Suicide history	0.908	0.240	14.331	2.479	(1.549, 3.967)	**<0.001**
ASLEC	0.019	0.005	16.121	1.019	(1.010, 1.029)	**<0.001**

ASLEC, adolescent self-rating life events checklist; CES-D, center for epidemiological studies depression scale; CI, confidence interval; OR, odds ratio.

## Discussion

Research on NSSI among adolescents has focused on the general population, so much less is known about its prevalence and drivers among adolescent psychiatric inpatients (21). Even less is known about these questions within the framework of the recently published DSM-5 criteria for diagnosing NSSI. Our study of 505 adolescent patients in the psychiatric ward of a large, comprehensive medical center in China revealed a disturbingly high NSSI prevalence of 77.8%, indicating that NSSI behavior was widespread in the clinical sample of adolescents with psychiatric disorders. While our univariate analysis suggested that numerous clinicodemographic factors may influence risk of NSSI, our multivariate analysis singled out female gender, younger age (10–14 years), depression symptoms, suicide history, and higher ASLEC scores as independent predictors of NSSI. These findings may help develop effective interventions to reduce NSSI among adolescent inpatients.

Influenced by research methods, cultural background, NSSI definition and other factors, NSSI incidence rate in each study are different. Previous studies (3, 41) have shown that the incidence of NSSI from hospital samples is more than 40.0%, and that from community samples is about 20.0%. A study of the Germany found that 37% (*N* = 41) of hospitalized adolescent psychiatric patients (*N* = 111) met the criteria for NSSI disorders according to DSM-5 criteria (42). Another study reported that 198 adolescents with psychiatric disorders at a hospital in the eastern United States and found that about 50% (*N* = 98) of the subjects met the DSM-5 criteria for NSSI disorders (43). The particular high prevalence in our study may reflect the problem of psychiatric adolescent inpatients’ NSSI behavior is becoming more serious and should not be underestimated and the possibility may be due to at least two factors. First, NSSI occurs in the context of a psychopathological disorder, and second, NSSI is ‘contagious’ in clinical contexts (especially in the hospitalization setting) (44).

Female adolescents in our sample were at greater risk of NSSI than male adolescents, consistent with studies of adolescents in the general population (22, 45). Female adolescents who suffer depression or anxiety may be more likely to engage in NSSI to relieve negative emotions (46). We also found that younger adolescents were at greater risk of NSSI than older ones, consistent with reports that the frequency of NSSI episodes peaks in mid-adolescence and declines in late adolescence (10, 47).

Family environment plays an important role in shaping the behaviors of children and adolescents (48). In this study, we found that parental scolding was associated with greater risk of NSSI. Harsh authoritarian parenting has been linked to greater risk of NSSI among adolescents (46, 47), and associations have been proposed among poor family functioning, NSSI, depression symptoms (49) and use of avoidance/emotion-focused coping (50). The available evidence suggests that harsh parenting can lead adolescents to engage in NSSI in order to punish themselves (51). According to Attachment Theory, the parent–child relationship is the primary relationship that a child has and the health of the relationship influences child’s social and emotional development (48). The development of intervention measures for NSSI among Chinese adolescents in home, school settings or hospital should consider positive parenting styles, which are more supportive, less psychologically controlling and reactive/punitive (52). Furthermore, the greater learning burden was associated with greater risk of NSSI. Under greater learning pressure, adolescent patients are more likely to experience academic anxiety and burnout characterized by insecurity, avoidance and procrastination (53), because their self-regulation ability is limited, they may resort to self-injury in order to mitigate the stress (54). Thus, education departments should advocate for reducing adolescents’ learning burden, and multi-level interventions could be implemented at schools for adolescents to help strengthen their learning skills.

We found that most of our participants had a history of suicide, consistent with analysis of other psychiatric adolescent inpatients (55). In addition, in the psychiatric inpatient environment, the NSSI detection rate among adolescents with different diseases varies, which may be related to the different degrees of emotional symptoms caused by a specific pathology. Future research should explore the prevalence and risk factors of NSSI among adolescents with different psychiatric disorders.

Our Chinese adolescent sample reported that their NSSI behaviors were motivated most often by the need to influence their surroundings or vent negative emotions, and less often by a search for stimulation (56). We found that NSSI was associated with negative life events and with symptoms of anxiety and depression, consistent with another study of adolescent psychiatric inpatients (57, 58). This is consistent with the idea that the combination of life stress, intrapersonal risk factors and difficulties with interpersonal communication increase risk of NSSI (23). The reason may be that NSSI behaviors and depressive episodes can operate in a reinforcing manner, such that NSSI provides relief from the negative affect experienced during depressive episodes (59). At the same time, adolescents with anxiety and depression usually face difficulties in emotional regulation. Patients are in a high frequency of negative emotional state and have low tolerance to emotion. When faced with emotional distress, difficulties in understanding and accepting emotion and suppressing impulsive behavior may lead to NSSI (60).

Indeed, we found interpersonal relationships, learning pressure, and sense of punishment, healthy adaptation, and other factors to be worse among our inpatients with NSSI than among those who did not report NSSI. According to the construction of ASLEC, interpersonal relationships include “being misunderstood, discrimination, quarrelling with classmates and friends, and losing face in public.” Interpersonal relationships during school have important influence on students’ cognitive, emotional, and personality development, and may even exceed family influence (61). Poor interpersonal relationships can lead to mental health problems in adolescents (58). According to the theory of the intentional self-injury emotion avoidance model, affective factors (such as interpersonal conflict) lead to strong adverse effects, combine with the defective emotional management mechanism, and then lead to NSSI (62). Previous study has confirmed that interpersonal stress was a significant mediator in the relationship between childhood betrayal trauma and depressive symptoms (63). Children and youth who were exposed to some form of interpersonal trauma were more likely to have mental health issues requiring urgent follow-up service compared to those who were not exposed (64). The main task of adolescents in school is to learn, and China is under great pressure to study and is highly competitive, long-term excessive learning pressure can lead to anger, anxiety, helplessness, depression, shame and boredom (61). These excessive and unnecessary emotional experiences lead to adolescents’ emotional management disorders, leading to the occurrence of NSSI behaviors (61). “Health adaptation factor” includes: significant changes in living habits, dislike of going to school, broken relationships, long-term distance from family and tense relationship with teachers. The degree of health adaptation of psychiatric inpatients with NSSI behavior is higher than that of non-NSSI group, and there is obvious health maladjustment. In addition, one factor that has to be considered is the occurrence of NSSI may be more common during and after COVID-19 pandemic due to COVID-19 related social distance and school lockdown policies, and isolation (30).

Our findings suggest that interventions aimed at promoting productive coping strategies for adverse life events such as stressful interpersonal relationships, romantic difficulties, and academic pressure may reduce risk of NSSI among psychiatric inpatients. Interventions should also treat symptoms of depression and anxiety.

Our study provides one of the few available analyses of NSSI among adolescent psychiatric inpatients, based on DSM-5 criteria. And we collected our data during face-to-face interviews, which may have increased data reliability. On the other hand, our findings should be interpreted with caution given that our sample came from a single center, so the representativeness of the sample might be limited. And the data came from self-report, increasing risk of recall bias and self-censure because of social norms around self-injury. In addition, our study is a cross-sectional study design and cannot verify causal relationships. Future work should verify and extend our findings by examining inpatient populations at multiple centers with a longitudinal design.

## Conclusion

This study appears to be one of the few studies that examine factors associated with NSSI, diagnosed according to DSM-5 criteria, among Chinese adolescents in a psychiatric ward. The high incidence of NSSI among this population is concerning, and treating it effectively may require reinforcing coping with adverse life events and mitigating symptoms of anxiety and depression. A better understanding of this behavior and its associated factors may inform interventions to reduce it.

## Data availability statement

The raw data supporting the conclusions of this article will be made available by the authors, without undue reservation.

## Ethics statement

The studies involving human participants were reviewed and approved by the Biomedical Research Ethics Committee of the West China Hospital of Sichuan University (approval 20201049). Written informed consent to participate in this study was provided by the participants’ legal guardian/next of kin.

## Author contributions

QX and XS designed the study and wrote the manuscript, which XH reviewed. LH and DH contributed to the implementation of the study. All authors contributed to the article and approved the submitted version.

## Funding

This research was funded by the Key Research and Development Projects of Sichuan Provincial Department of Science and Technology (grant no. 2021YFS0151), the Popular Science Training Program of Sichuan Provincial Department of Science and Technology (grant no. 2022JDKP0068), the Scientific Research Project of Sichuan Provincial Health Commission (Popularization and Application Project, grant no. 20PJ014), the Project of Chengdu Science and Technology Bureau (2021-YF05-02173-SN), the West China Nursing Discipline Development Special Fund Project, Sichuan University (grant no. HXHL21022), and the Sichuan Nursing Scientific Research Project Plan (grant no. H20009).

## Conflict of interest

The authors declare that the research was conducted in the absence of any commercial or financial relationships that could be construed as a potential conflict of interest.

The reviewer CQ declared a shared affiliation with the authors to the handling editor at the time of review.

## Publisher’s note

All claims expressed in this article are solely those of the authors and do not necessarily represent those of their affiliated organizations, or those of the publisher, the editors and the reviewers. Any product that may be evaluated in this article, or claim that may be made by its manufacturer, is not guaranteed or endorsed by the publisher.

## References

[1] Vega D Sintes A Fernández M Puntí J Soler J Santamarina P. Review and update on non-suicidal self-injury: who, how and why? Actas Esp Psiquiatr. (2018) 46:146–55. PMID: 30079928

[2] Zetterqvist M. The DSM-5 diagnosis of nonsuicidal self-injury disorder: a review of the empirical literature. Child Adolesc Psychiatry Ment Health. (2015) 9:31. doi: 10.1186/s13034-015-0062-7, PMID: 26417387 PMC4584484

[3] Hauber K Boon A Vermeiren R. Non-suicidal self-injury in clinical practice. Front Psychol. (2019) 10:502. doi: 10.3389/fpsyg.2019.00502, PMID: 30930814 PMC6424099

[4] Huang FJ Liu TB. Association between non-suicidal self-injury behavior and impulsivity in adolescent patients with depressive disorder in the first hospitalization. Sichuan Mental Health. (2022) 35:132–6. doi: 10.11886/scjsws20211208001

[5] Halicka J Kiejna A. Non-suicidal self-injury (NSSI) and suicidal: criteria differentiation. Adv Clin Exp Med. (2018) 27:257–61. doi: 10.17219/acem/66353, PMID: 29521070

[6] Ulloa Flores RE Mayer Villa PA de la Peña OF Palacios Cruz L Victoria FG. Lesiones autoinfligidas con fines no suicidas según el DSM-5 en una muestra clínica de adolescentes mexicanos con autolesiones. Revista Colombiana de Psiquiatría. (2020) 49:39–43. doi: 10.1016/j.rcp.2018.04.002 32081207

[7] Cipriano A Cella S Cotrufo P. Nonsuicidal self-injury: a systematic review. Front Psychol. (2017) 8:1946. doi: 10.3389/fpsyg.2017.01946, PMID: 29167651 PMC5682335

[8] Hepp J Carpenter RW Storkel LM Schmitz SE Schmahl C Niedtfeld I. A systematic review of daily life studies on non-suicidal self-injury based on the four-function model. Clin Psychol Rev. (2020) 82:101888. doi: 10.1016/j.cpr.2020.101888, PMID: 32949907 PMC7680364

[9] Wester KL Ivers N Villalba JA Trepal HC Henson R. The relationship between nonsuicidal self-injury and suicidal ideation. J Couns Dev. (2016) 94:3–12. doi: 10.1002/jcad.12057

[10] Brown RC Plener PL. Non-suicidal self-injury in adolescence. Curr Psychiatry Rep. (2017) 19:20. doi: 10.1007/s11920-017-0767-9, PMID: 28315191 PMC5357256

[11] Thai TT Jones MK Nguyen TP Pham TV Bui HHT Kim LX. The prevalence, correlates and functions of non-suicidal self-injury in Vietnamese adolescents. Psychol Res Behav Manag. (2021) 14:1915–27. doi: 10.2147/PRBM.S339168, PMID: 34866944 PMC8636691

[12] Jeong JY Kim DH. Gender differences in the prevalence of and factors related to non-suicidal self-injury among middle and high school students in South Korea. Int J Environ Res Public Health. (2021) 18:5965. doi: 10.3390/ijerph18115965, PMID: 34199442 PMC8199576

[13] Tang J Li G Chen B Huang Z Zhang Y Chang H. Prevalence of and risk factors for non-suicidal self-injury in rural China: results from a nationwide survey in China. J Affect Disord. (2018) 226:188–95. doi: 10.1016/j.jad.2017.09.051, PMID: 28988001

[14] Esposito C Dragone M Affuso G Amodeo AL Bacchini D. Prevalence of engagement and frequency of non-suicidal self-injury behaviors in adolescence: an investigation of the longitudinal course and the role of temperamental effortful control. Eur Child Adolesc Psychiatry. (2022). doi: 10.1007/s00787-022-02083-7, PMID: 36123505 PMC10682258

[15] Xiao Q Song X Huang L Hou D Huang X. Global prevalence and characteristics of non-suicidal self-injury between 2010 and 2021 among a non-clinical sample of adolescents: a meta-analysis. Front Psych. (2022) 13:912441. doi: 10.3389/fpsyt.2022.912441, PMID: 36032224 PMC9399519

[16] Kaess M Parzer P Mattern M Plener PL Bifulco A Resch F. Adverse childhood experiences and their impact on frequency, severity, and the individual function of nonsuicidal self-injury in youth. Psychiatry Res. (2013) 206:265–72. doi: 10.1016/j.psychres.2012.10.012, PMID: 23159195

[17] Wang L Liu J Yang Y Zou H. Prevalence and risk factors for non-suicidal self-injury among patients with depression or bipolar disorder in China. BMC Psychiatry. (2021) 21:389. doi: 10.1186/s12888-021-03392-y, PMID: 34348675 PMC8335871

[18] Plener PL. Non-suicidal self-injury as autonomous diagnosis—implications for research and clinic of the DSM-5 proposal to establish the diagnosis of non-suicidal self-injury in adolescents. Zeitschrift fur Kinder-und Jugendpsychiatrie und Psychotherapie. (2012) 40:113–20. doi: 10.1024/1422-4917/a000158, PMID: 22354495

[19] Somma A Fossati A Ferrara M Fantini F Galosi S Krueger RF. DSM-5 personality domains as correlates of non-suicidal self-injury severity in an Italian sample of adolescent inpatients with self-destructive behaviour. Personal Ment Health. (2019) 13:205–14. doi: 10.1002/pmh.1462, PMID: 31353830

[20] Gander M Fuchs M Franz N Jahnke-Majorkovits A-C Buchheim A Bock A. Non-suicidal self-injury and attachment trauma in adolescent inpatients with psychiatric disorders. Compr Psychiatry. (2021) 111:152273. doi: 10.1016/j.comppsych.2021.152273, PMID: 34555553

[21] Zetterqvist M Lundh L-G Dahlström Ö Svedin CG. Prevalence and function of non-suicidal self-injury (NSSI) in a community sample of adolescents, using suggested DSM-5 criteria for a potential NSSI disorder. J Abnorm Child Psychol. (2013) 41:759–73. doi: 10.1007/s10802-013-9712-5, PMID: 23344701

[22] Ghinea D Edinger A Parzer P Koenig J Resch F Kaess M. Non-suicidal self-injury disorder as a stand-alone diagnosis in a consecutive help-seeking sample of adolescents. J Affect Disord. (2020) 274:1122–5. doi: 10.1016/j.jad.2020.06.009, PMID: 32663940

[23] Liu RT Cheek SM Nestor BA. Non-suicidal self-injury and life stress: a systematic meta-analysis and theoretical elaboration. Clin Psychol Rev. (2016) 47:1–14. doi: 10.1016/j.cpr.2016.05.005, PMID: 27267345 PMC4938721

[24] Baetens I Greene D Van Hove L Van Leeuwen K Wiersema JR Desoete A. Predictors and consequences of non-suicidal self-injury in relation to life, peer, and school factors. J Adolesc. (2021) 90:100–8. doi: 10.1016/j.adolescence.2021.06.005, PMID: 34182197

[25] Wang Q Liu X. Peer victimization, depressive symptoms and non-suicidal self-injury behavior in Chinese migrant children: the roles of gender and stressful life events. Psychol Res Behav Manag. (2019) 12:661–73. doi: 10.2147/PRBM.S215246, PMID: 31496850 PMC6698170

[26] Gao Y Wang H Liu X Xiong Y Wei M. Associations between stressful life events, non-suicidal self-injury, and depressive symptoms among Chinese rural-to-urban children: a three-wave longitudinal study. Stress Health. (2020) 36:522–32. doi: 10.1002/smi.2954, PMID: 32369249

[27] Yang L Sun L Zhang Z Sun Y Wu H Ye D. Internet addiction, adolescent depression, and the mediating role of life events: finding from a sample of Chinese adolescents. Int J Psychol. (2014) 49:342–7. doi: 10.1002/ijop.12063, PMID: 25178955

[28] Corrieri S Heider D Conrad I Blume A Konig HH Riedel-Heller SG. School-based prevention programs for depression and anxiety in adolescence: a systematic review. Health Promot Int. (2013) 29:427–41. doi: 10.1093/heapro/dat001 23376883

[29] Chen H Guo H Chen H Cao X Liu J Chen X. Influence of academic stress and school bullying on self-harm behaviors among Chinese middle school students: the mediation effect of depression and anxiety. Frontiers. Public Health. (2023) 10:10. doi: 10.3389/fpubh.2022.1049051 PMC9853286 36684901

[30] Jiao T Guo S Zhang Y Li Y Xie X Ma Y. Associations of depressive and anxiety symptoms with non-suicidal self-injury and suicidal attempt among Chinese adolescents: the mediation role of sleep quality. Frontiers. Psychiatry. (2022) 13:13. doi: 10.3389/fpsyt.2022.1018525 PMC9814715 36620676

[31] Liu XC Liu LQ Yang J Cai FX Wang AZ Sun LM. Establishment and reliability and validity test of adolescent life events scale. Shangdong Psychiatr. (1997) 10:15–9.

[32] Nock MK. Self-injury. Annu Rev Clin Psychol. (2010) 6:339–63. doi: 10.1146/annurev.clinpsy.121208.131258 20192787

[33] Wei C Wang Y Ma T Zou Q Xu Q Lu H. Gratitude buffers the effects of stressful life events and deviant peer affiliation on adolescents' non-suicidal self-injury. Front Psychol. (2022) 13:939974. doi: 10.3389/fpsyg.2022.939974, PMID: 36248536 PMC9561820

[34] Gandhi A Luyckx K Goossens L Maitra S Claes L. Association between non-suicidal self-injury, parents and peers related loneliness, and attitude towards aloneness in Flemish adolescents: an empirical note. Psychol Belgica. (2018) 58:3–12. doi: 10.5334/pb.385, PMID: 30479803 PMC6194522

[35] Sornberger MJ Heath NL Toste JR McLouth R. Nonsuicidal self-injury and gender patterns of prevalence, methods, and locations among adolescents. Suicide Life Threat Behav. (2012) 42:266–78. doi: 10.1111/j.1943-278X.2012.0088.x 22435988

[36] Victor SE Davis T Klonsky ED. Descriptive characteristics and initial psychometric properties of the non-suicidal self-injury disorder scale. Arch Suicide Res. (2017) 21:265–78. doi: 10.1080/13811118.2016.1193078, PMID: 27267416 PMC5832024

[37] Han Y Fan J Wang X Xia J Liu X Zhou H. Factor structure and gender invariance of Chinese version state-trait anxiety inventory (form Y) in university students. Front Psychol. (2020) 11:2228. doi: 10.3389/fpsyg.2020.02228, PMID: 33132946 PMC7578736

[38] Liu Z Li X Ge X. Left too early: the effects of age at separation from parents on Chinese rural children's symptoms of anxiety and depression. Am J Public Health. (2009) 99:2049–54. doi: 10.2105/AJPH.2008.150474, PMID: 19762669 PMC2759782

[39] Radloff LS. The CES-D scale—a self-report depression scale for research in the general population. Appl Psychol Meas. (1977) 1:385–401. doi: 10.1177/014662167700100306

[40] Vilagut G Forero CG Barbaglia G Alonso J. Screening for depression in the general population with the Center for Epidemiologic Studies Depression (CES-D): a systematic review with Meta-analysis. PLoS One. (2016) 11:e0155431. doi: 10.1371/journal.pone.0155431, PMID: 27182821 PMC4868329

[41] Glenn CR Lanzillo EC Esposito EC Santee AC Nock MK Auerbach RP. Examining the course of suicidal and nonsuicidal self-injurious thoughts and behaviors in outpatient and inpatient adolescents. J Abnorm Child Psychol. (2017) 45:971–83. doi: 10.1007/s10802-016-0214-0, PMID: 27761783 PMC5397367

[42] Groschwitz RC Kaess M Fischer G Ameis N Schulze UME Brunner R. The association of non-suicidal self-injury and suicidal behavior according to DSM-5 in adolescent psychiatric inpatients. Psychiatry Res. (2015) 228:454–61. doi: 10.1016/j.psychres.2015.06.019, PMID: 26144578

[43] Glenn CR Klonsky ED. Nonsuicidal self-injury disorder: an empirical investigation in adolescent psychiatric patients. J Clin Child Adolesc Psychol. (2013) 42:496–507. doi: 10.1080/15374416.2013.794699, PMID: 23682597 PMC4433043

[44] Syed S Kingsbury M Bennett K Manion I Colman I. Adolescents’ knowledge of a peer’s non-suicidal self-injury and own non-suicidal self-injury and suicidality. Acta Psychiatr Scand. (2020) 142:366–73. doi: 10.1111/acps.13229, PMID: 32885408

[45] Bresin K Schoenleber M. Gender differences in the prevalence of nonsuicidal self-injury: a meta-analysis. Clin Psychol Rev. (2015) 38:55–64. doi: 10.1016/j.cpr.2015.02.009 25795294

[46] Hu CC Jl H Shang YS Huang TT Lin J Xie J. Effects of emotional dysregulation on nonsuicidal self-injury in adolescents with mood disorders. Zhejiang Med J. (2022) 44:1833–6. doi: 10.12056/j.issn.1006-2785.2022.44.17.2022-1492

[47] Wilkinson PO Qiu T Jesmont C Neufeld SAS Kaur SP Jones PB. Age and gender effects on non-suicidal self-injury, and their interplay with psychological distress. J Affect Disord. (2022) 306:240–5. doi: 10.1016/j.jad.2022.03.021, PMID: 35304237

[48] Liu Y Xiao Y Ran H He X Jiang L Wang T. Association between parenting and non-suicidal self-injury among adolescents in Yunnan, China: a cross-sectional survey. PeerJ. (2020) 8:e10493. doi: 10.7717/peerj.10493, PMID: 33354431 PMC7727394

[49] Baetens I Andrews T Claes L Martin G. The association between family functioning and NSSI in adolescence: the mediating role of depressive symptoms. Family Sci. (2015) 6:330–7. doi: 10.1080/19424620.2015.1056917

[50] Ren Y Lin MP Liu YH Zhang X Wu JY Hu WH. The mediating role of coping strategy in the association between family functioning and nonsuicidal self-injury among Taiwanese adolescents. J Clin Psychol. (2018) 74:1246–57. doi: 10.1002/jclp.22587, PMID: 29355974

[51] Ammerman BA Brown S. The mediating role of self-criticism in the relationship between parental expressed emotion and NSSI. Curr Psychol. (2018) 37:325–33. doi: 10.1007/s12144-016-9516-1, PMID: 29651223 PMC5891159

[52] Fong ZH Loh WNC Fong YJ Neo HLM Chee TT. Parenting behaviors, parenting styles, and non-suicidal self-injury in young people: a systematic review. Clin Child Psychol Psychiatry. (2021) 27:61–81. doi: 10.1177/13591045211055071 34866412

[53] Wang X Li X Guo C Hu Y Xia L Geng F. Prevalence and correlates of alexithymia and its relationship with life events in Chinese adolescents with depression during the COVID-19 pandemic. Front Psych. (2021) 12:774952. doi: 10.3389/fpsyt.2021.774952, PMID: 34880795 PMC8645693

[54] Ao C Hu W Zhou F Hu DZ Long XX Huang P. Analysis of differences between middle school and high school students’ help-seeking behaviors and association with nonsuicidal self-injury. Chin J Sch Health. (2021) 42:597–601. doi: 10.16835/j.cnki.1000-9817.2021.04.027

[55] Sevecke K Bock A Fenzel L Fuchs M Gander M. Nonsuicidal self-injury in a naturalistic sample of adolescents undergoing inpatient psychiatric treatment: prevalence. Gender Distribution and Comorbidities Psychiatria Danubina. (2017) 29:522–8. doi: 10.24869/psyd.2017.522 29197214

[56] Chen H Pan B Zhang C Guo Y Zhou J Wang X. Revision of the non-suicidal self-injury behavior scale for adolescents with mental disorder. J Cent South Univ (Med Sci). (2022) 47:301–8. doi: 10.11817/j.issn.1672-7347.2022.210549 PMC10930059 35545322

[57] Zhang JJ Liu YD Zhang H Huang ZH Wang F Yang JJ. Correlates of non-suicidal self-injury in adolescent psychiatric patients in China. Front Psych. (2022) 13:864150. doi: 10.3389/fpsyt.2022.864150, PMID: 35832596 PMC9271878

[58] Shao C Wang X Ma Q Zhao Y Yun X. Analysis of risk factors of non-suicidal self-harm behavior in adolescents with depression. Ann Palliative Med. (2021) 10:9607–13. doi: 10.21037/apm-21-1951, PMID: 34628886

[59] Millon EM Alqueza KL Kamath RA Marsh R Pagliaccio D Blumberg HP. Non-suicidal self-injurious thoughts and behaviors among adolescent inpatients. Child Psychiatry Hum Dev. (2022). doi: 10.1007/s10578-022-01380-1, PMID: 35727385 PMC9782727

[60] Serra M Presicci A Quaranta L Caputo E Achille M Margari F. Assessing clinical features of adolescents suffering from depression who engage in non-suicidal self-injury. Children (Basel). (2022) 9:9. doi: 10.3390/children9020201 PMC8870312 35204921

[61] Yang F Jiang L Miao J Xu X Ran H Che Y. The association between non-suicidal self-injury and negative life events in children and adolescents in underdeveloped regions of South-Western China. PeerJ. (2022) 10:e12665. doi: 10.7717/peerj.12665, PMID: 35287346 PMC8917796

[62] Chapman AL Gratz KL Brown MZ. Solving the puzzle of deliberate self-harm: the experiential avoidance model. Behav Res Ther. (2006) 44:371–94. doi: 10.1016/j.brat.2005.03.005, PMID: 16446150

[63] Fung HW Lam SKK Chien WT Hung SL Ling HW-H Lee VWP. Interpersonal stress mediates the relationship between childhood trauma and depressive symptoms: findings from two culturally different samples. Aust N Z J Psychiatry. (2022):000486742211385. doi: 10.1177/00048674221138501, PMID: 36440622

[64] Marshall C Semovski V Stewart SL. Exposure to childhood interpersonal trauma and mental health service urgency. Child Abuse Negl. (2020) 106:104464. doi: 10.1016/j.chiabu.2020.104464, PMID: 32497938

